# Rare malignant spindle cell sarcoma of the left atrium diagnosed with TEE: A case report

**DOI:** 10.1097/MD.0000000000024033

**Published:** 2021-03-12

**Authors:** Yuan-Yuan Sun, Xin-yu Wang, Guo-Ming Zhang, Xu Chen, Bo Jing, Yuan Wu, Yu Song, Mao-Long Su

**Affiliations:** a Department of Ultrasound; b Department of Cardiology; c Department of cardiac surgery, Xiamen Cardiovascular Hospital Xiamen University, Xiamen, China.

**Keywords:** case report, malignant spindle cell sarcoma, transesophageal echocardiography

## Abstract

**Introduction:**

One of the purposes of echocardiography is to determine the nature of a space-occupying lesion. The conventional transthoracic echocardiogram (TTE) is the preferred method for the diagnosis of cardiac space-occupying lesions as it can reveal the baseline information. For patients with poor conditions, however, TTE cannot clearly display the boundary, it has a limited role in determining the nature of the lesions.

**Patient concerns:**

A 47-year-old woman presented with intermittent fever for 7 days and chest distress/shortness of breath for 5 days.

**Diagnosis:**

In our current case, we inferred the nature of space-occupying lesions in the left atrium more accurately using transesophageal echocardiography (TEE) than TTE, which may offer diagnostic evidence for surgical treatment.

**Interventions:**

The patient underwent surgical resection of the left atrial tumor and reconstruction of the left atrial wall. However, the patient's posterior lobe of the mitral valve was infiltrated by tumor, which was difficult to completely remove.

**Outcomes:**

Echocardiography was performed 3 months after surgery and the tumor recurred in the posterior lobe of the mitral valve. Although almost all tumors have been removed by surgery, the average survival time is often less than 1 year, as it is difficult to completely remove and easy to relapse with poor prognosis.

**Conclusion:**

Transesophageal echocardiography (TEE) plays a relatively more important role in the determination and differential diagnosis of cardiac space-occupying lesions

## 1 Introduction

Space-occupying lesions of the heart mainly include benign and malignant tumors, thrombus and abscesses, although hematomas have also been reported. Surgical resection is the mainstay treatment for benign tumors, whereas surgery, chemotherapy, and/or radiotherapy can be chosen for the treatment of malignant tumors depending on their pathological type and disease status.^[1]^ Thus, identifying the nature of a mass is a prerequisite for rational treatment selection. The conventional transthoracic TTE is the preferred method for the diagnosis of cardiac space-occupying lesions as it can reveal the baseline information, such as location, size and morphology. For patients with poor conditions, however, TTE cannot clearly display the boundary, root and pedicles of the tumor and its relationships with surrounding tissues; in addition, it has a limited role in determining the nature of the lesions. Transesophageal echocardiography(TEE) can provide clearer images and clarify the nature of space-occupying lesions, which has a great advantage over TTE.^[2,3]^ In our current case, we inferred the nature of space-occupying lesions more accurately using TEE than TTE, which may offer diagnostic evidence for surgical treatment.

## 2 Patient information

### 2.1 Chief complaints

A 47-year-old woman presented with intermittent fever for 7 days and shortness of breath for 5 days.

### 2.2 History of past illness

She was generally healthy but had a history of hypertension for 20 years. She denied any history of diabetes, hepatitis, or infectious disease (e.g., tuberculosis). She had no history of allergy, blood transfusion, trauma, or surgery. The relevant physical examination (PE) results showed no abnormality.

## 3 Diagnostic assessment

### 3.1 Laboratory examinations

D-dimer was 2.36 mg/L (reference range: 0–0.55 mg/L); white blood cell count, 12.3 × 10^9^/L; neutrophil ratio, 84.3%; NT-proBNP’, 1284 pg/ml (reference range: 0–125 pg/ml); procalcitonin (PCT), 0.07 ng/ml (reference range: 0–0.05 ng/ml); tumor marker CA-125, 173.8 U/ml (reference range: 0–49 U/ml); and neuron-specific enolase (NSE), 27.45 ng/ml (reference range: 0–16 ng/ml).

### 3.2 Imaging examinations

**Figure 1:**
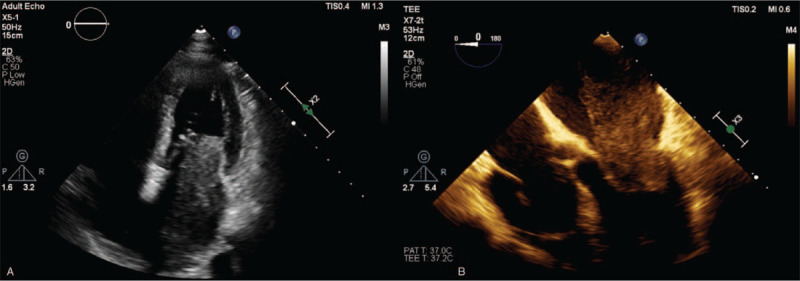
Compared with Routine TTE(A), TEE(B) was able to completely present the size, irregular shape, markedly heterogeneous internal echoes, broad base, involving the left atrial wall and the posterior leaflet of the mitral valve with a wide range and unclear boundary and adhesion of the mass in the same section (Apical four-chamber heart section).

**Figure 2:**
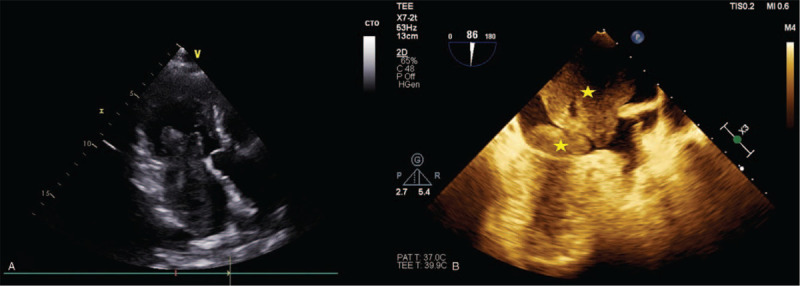
Compared with Routine TTE (A), TEE (B) could display the exact number of tumors (2, yellow stars) in the same section(Apical three-chamber heart section). The masses had no clear boundary with the endocardium or pericardium with low mobility.

**Figure 3:**
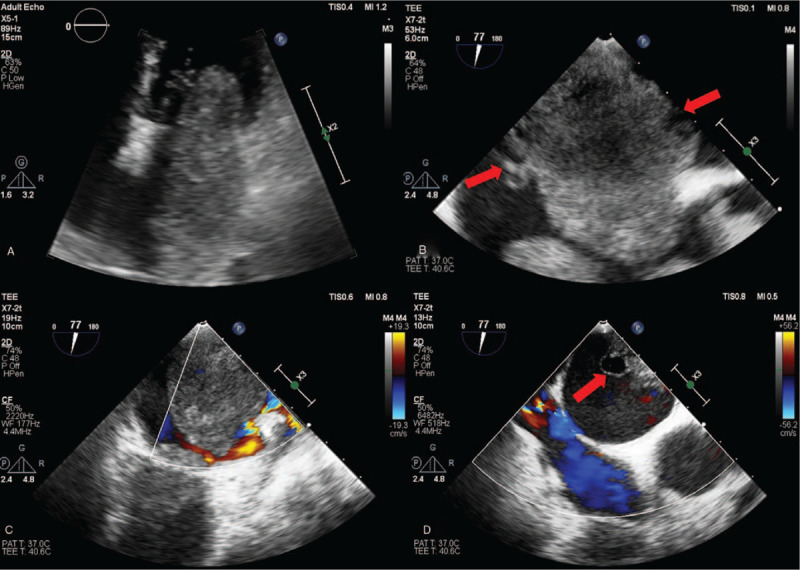
Compared with Routine TTE (A), TEE (B,C,D) could display the internal echoes of the tumor more clearly due to the high frequency and high resolution of its probe, involving multiple vesicle-like changes on the tumor surface(red arrows), which may be specific to malignant tumors.

Routine TTE revealed 2 dense-echo masses (5.5 cm × 2.7 cm and 3.4 cm × 1.7 cm in size, respectively) in the left atrium. The masses showed irregular shapes, and markedly heterogeneous internal echoes. The masses had poor mobility and were tightly attached to the posterior mitral valve, accompanied by pericardial effusion (Figs. 1–3).

TEE showed 2 dense-echo masses in the left atrium. One mass was large in size and had irregular shape, heterogeneous internal echoes, and broad base, involving the left atrial wall and the posterior leaflet of the mitral valve, with a wide range and unclear boundary and adhesion. It had no clear boundary with the endocardium or pericardium. The masses had low mobility and were accompanied by a small amount of pericardial effusion (Figs. 1–3).

**Figure 4:**
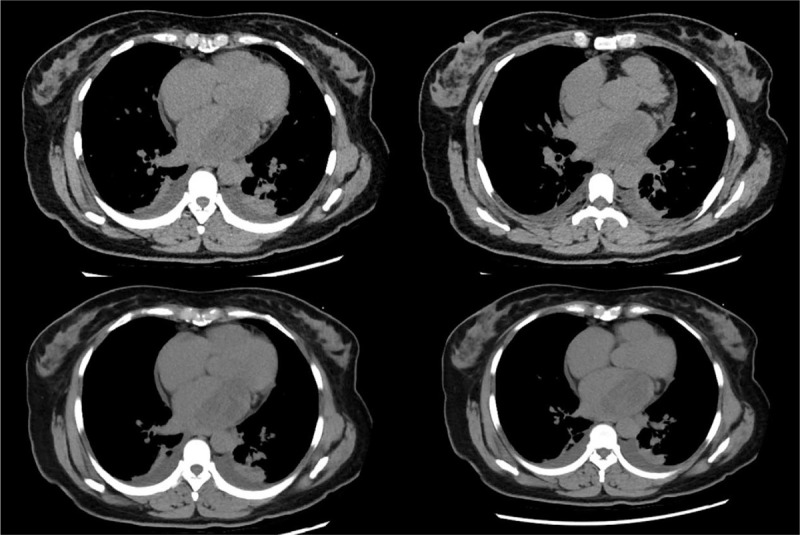
CT scanning showed an increase in the volume of the left atrium; a slightly low-dense patchy mass was visible inside the left atrium, with a CT value of about 25 HU.

CT scanning showed an increase in the volume of the left atrium; a slightly low-dense patchy mass was visible inside the left atrium, with a CT value of about 25 HU (Fig. 4).

## 4 Therapeutic intervention

### 4.1 Treatment and final diagnosis

**Figure 5:**
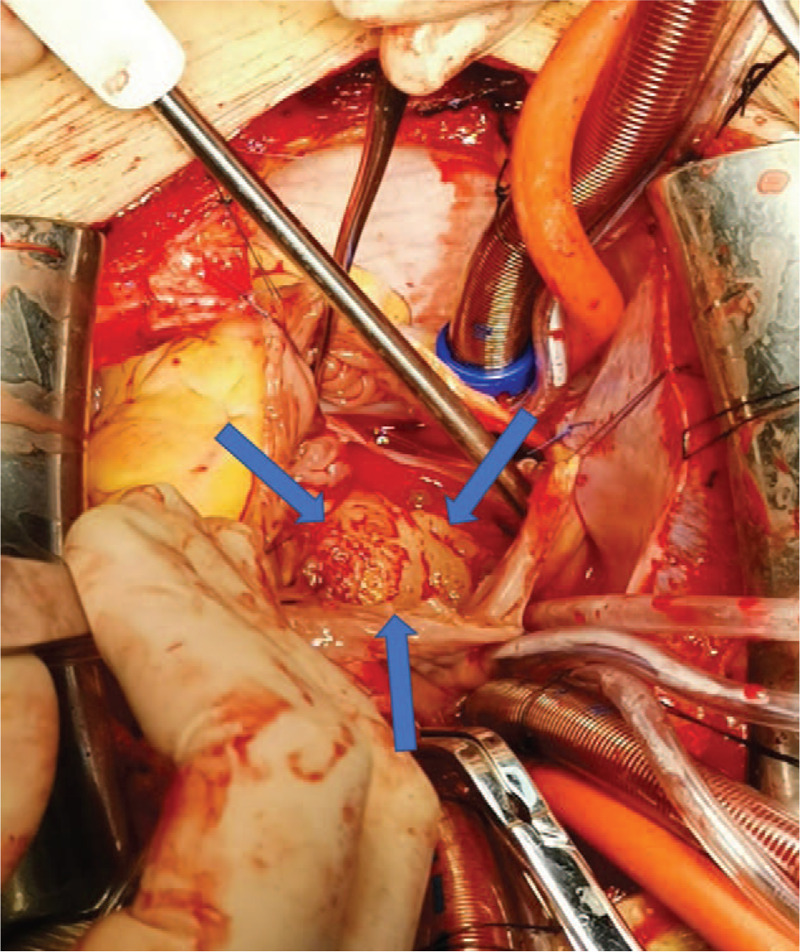
During the operation, when the pericardium was opened, morphologically, the tumor had a yellow jelly-like appearance (Blue arrows).

**Figure 6:**
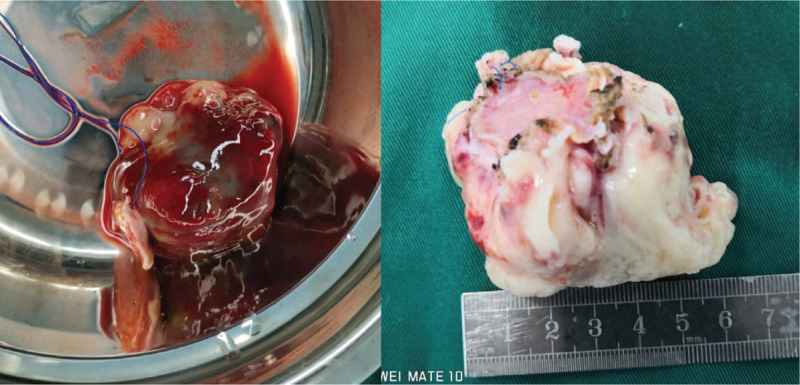
The larger tumor of 6 cm × 5 cm × 3 cm with a broad base tumor was fish-meat like when cut open.

**Figure 7:**
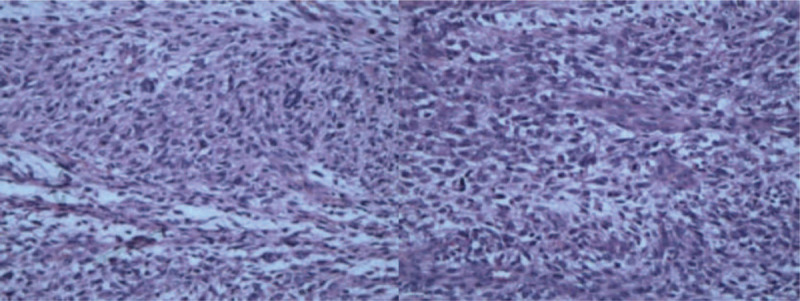
Pathology confirmed the diagnosis of a spindle cell sarcoma in the left atrium.

**Figure 8:**
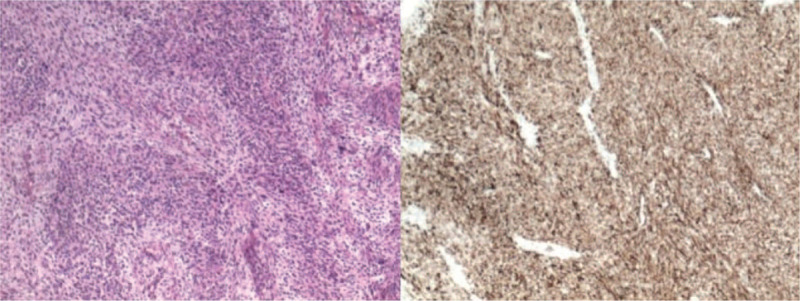
Immunohistochemical examination was also performed, which result was endocardial sarcoma (differentiation into smooth muscle): Muc-4(−), SMA(+), Des(+), Myo-D1(+), S-100(−), CK-19(−), CD-34(−), Ki-67(40%).

The patient underwent surgical resection of the space-occupying lesions in the left atrium and reconstruction of the left atrial wall. During the operation, a tumor of 1.5 cm × 1.5 cm was seen at the top of the left atrium. In addition, a large tumor of 6 cm × 5 cm × 3 cm with a broad base was also found, whose pedicle was located in the posterior wall of left atrium and extended to the opening of left lower pulmonary vein. The posterior leaflet of mitral valve was completely invaded by tumor tissues, along with mitral valve stenosis and incompetence. Palliative resection of the tumor was performed. Morphologically, the tumor had a yellow jelly-like appearance (Fig. 5) and was fish-meat like when cut open (Fig. 6). The tumor was fragile. Pathology confirmed the diagnosis of a spindle cell sarcoma in the left atrium (Fig. 7), and immunohistochemical examination was also performed, which result was endocardial sarcoma (differentiation into smooth muscle) (Fig. 8).

## 5 Fellow-up and outcomes

**Figure 9:**
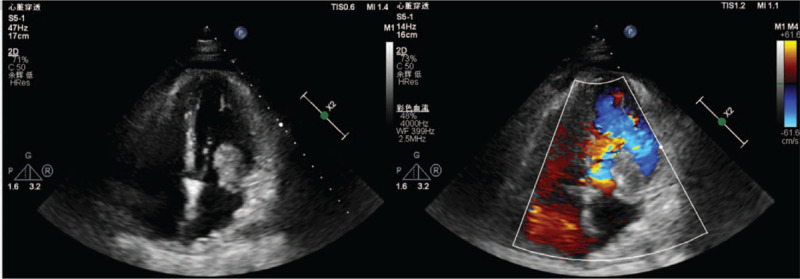
Echocardiography was performed 3 months after surgery and the tumor recurred in the posterior lobe of the mitral valve.

Echocardiography was performed 3 months after surgery and the tumor recurred in the posterior lobe of the mitral valve (Fig. 9). Although almost all tumors have been removed by surgery, the average survival time is often less than 1 year, as it is difficult to completely remove and easy to relapse with poor prognosis.

### 5.1 Timeline

???.

## 6 Discussion

Primary cardiac malignant tumors are rare, with an incidence of only 0.001% to 0.28%.^[4]^ The incidence of cardiac metastatic tumors is 20 to 40 times that of primary cardiac malignancies. Primary cardiac malignancies can occur in any part of the heart, but more frequently found in the right cardiac system, especially the right atrium.^[5]^ Cardiac sarcomas in the left atrium are often misdiagnosed as myxomas.^[6]^ Histologically, cardiac sarcomas can be classified into angiosarcoma, rhabdomyosarcoma, leiomyosarcoma, fibrosarcoma, malignant fibrohistiocytoma, and undifferentiated sarcomas. Cardiac spindle cell sarcoma is classified as undifferentiated sarcoma.^[7]^ It usually involves the left atrium and may extend into the pericardium, leading to pericardial hemorrhage. In contrast, cardiac angiosarcoma mostly occurs in the right atrium.^[8]^ The clinical manifestations of the primary spindle cell sarcoma of the left atrium are atypical. There may be no specific changes on conventional ECG and chest X-ray examinations. Echocardiography can often establish the diagnosis of a cardiac tumor. Ultrasound can reveal the location, morphology, and size of a cardiac tumor and differentiate the nature (benign or malignant) of the tumor. However, echocardiography is a subjective technique and is highly dependent on the experience of the ultrasonographist.

Currently, cardiac space-occupying lesions are usually discovered first by TTE, which can distinguish thrombosis from tumors. For tumors with typical ultrasound characteristics, two-dimensional ultrasound is sufficient to make a diagnosis.^[8,9]^ However, TTE often fails to make a correct diagnosis for cardiac tumors if the patient has poor body shape and inappropriate position or if the lesion is behind a rib. In our center, we once misdiagnosed a mass in the right atrium of an elderly patient as a myxoma by using TTE; later, the patient underwent TEE, which not only showed a lesion in the right atrium but also revealed multiple similar hyper-dense masses at the right atrial appendage wall and the superior vena cava entrance via multi-angle scanning. The final diagnosis by TEE was thrombosis. The lesion markedly shrank after thrombolytic therapy.

Compared with the conventional TTE, TEE has the following advantages:

1. TEE can more clearly display small structures such as tumor pedicles and small thrombi, which enables a definite diagnosis in combination with other imaging features and medical histories.^[8]^
2. As the TEE probe is closely attached to the left atrium with a higher frequency, TEE can clearly show the boundary between the space-occupying lesion and the surrounding tissues. A clear boundary between a lesion and its surrounding tissue is an important factor for distinguishing benign tumors from malignant ones.^[9]^
3. TEE is helpful in differentiating normal from abnormal structures. For example, located in front of the entrance of the superior and inferior vena cava, the crista terminalis of the right atrium is a fibrous muscular bridge that separates the primitive atrium from the sinus venarum cavarum. It has different morphologies depending on the degree of degradation.^[9,10]^ In a few patients, the crista terminalis protrudes from the cardiac cavity, which may be easily misdiagnosed as a space-occupying lesion on TTE; in contrast, TEE can reveal the connection of the base of this structure with the pectinate muscle of the right atrium, thus ruling out the diagnosis of a space-occupying lesion.
4. Compared with TTE, TEE can display the internal echoes of the tumor more clearly due to the high frequency and high resolution of its probe.^[10]^ These advantages enable TEE to make relatively more accurate diagnosis of the space-occupying lesions with typical sonographic changes.

In our current case, the nature of the mass was more accurately identified by TEE, which provided the surgeon with adequate preoperative information and assisted in formulating a reasonable surgical plan. Surgical pathology also showed that the mass had a poorly-demarcated boundary with its surrounding tissues; in particular, the tumor had infiltrated the posterior leaflet of the mitral valve and was difficult to be completely removed. Literature has shown that although surgical resection is the primary treatment for spindle cell sarcomas of the left atrium, these tumors are difficult to be completely removed and can easily recur. The prognosis is poor, with an average survival of less than 1 year.

In summary, although the incidence of cardiac malignancies is low, more accurate diagnosis and timely treatment are of great clinical importance. The TEE probe is placed very close to the left atrium and not affected by unfavorable factors such as ribs, thoracic gas, and obesity. In addition, as the TEE probe has a higher frequency than the TTE probe, it can more clearly display the tumor pedicle, small thrombus, thickened crista terminalis, and other structures that TTE does not easily show clearly. TEE can also demarcate the boundary between a space-occupying lesion and its adjacent tissues more clearly. Therefore, TEE is relative more superior over TTE in terms of diagnostic accuracy and can assist in formulating a surgical plan, and has higher value for diagnosis of malignant tumors.

## Author contributions

Sun YY, Chen X and Jing B acquired case information; Su ML designed research; Wang XY, Wu Y and Song Y acquired imaging examination data; and Sun YY and Zhang GM wrote the paper.

**Conceptualization:** Yuan-Yuan Sun, Guo-Ming Zhang, Xu Chen, Maolong Su.

**Data curation:** Yu Song, Maolong Su.

**Formal analysis:** Xin-yu Wang, Guo-Ming Zhang, Yuan Wu, Maolong Su.

**Funding acquisition:** Guo-Ming Zhang.

**Investigation:** Xin-yu Wang, Guo-Ming Zhang, Bo Jing, Maolong Su.

**Methodology:** Yuan-Yuan Sun, Xin-yu Wang, Xu Chen, Maolong Su.

**Resources:** Yuan-Yuan Sun, Xin-yu Wang, Bo Jing, Yuan Wu, Maolong Su.

**Software:** Guo-Ming Zhang, Yu Song.

**Validation:** Xin-yu Wang, Xu Chen, Maolong Su.

**Visualization:** Guo-Ming Zhang, Maolong Su.

**Writing – original draft:** Yuan-Yuan Sun, Guo-Ming Zhang.

**Writing – review & editing:** Yuan-Yuan Sun, Guo-Ming Zhang, Maolong Su.
